# Supplementary material for: Recognition of Maize Phenology in Sentinel Images with Machine Learning
Source: Sensors (Basel). 2021 Dec 24;22(1):94. doi: 10.3390/s22010094 (PMC8747376; doi:10.3390/s22010094)
Supplement: Supplementary file 1 [file sensors-22-00094-s001.zip › sensors-1450732 - Supplementary.pdf]

sensors

MDPI

Recognition of Maize Phenology in Sentinel Images with Machine Learning

Alvaro Murguia-Cozar [1], Antonia Macedo-Cruz [1,*], Demetrio Salvador Fernandez-Reynoso [1] and Jorge Arturo Salgado Transito [2]

[1] Colegio de Postgraduados, Campus Montecillo, Carretera federal Mexico-Texcoco, km. 36.5, Montecillo, Texcoco 56230, State of Mexico, Mexico; murguia.alvaro@colpos.mx (A.M.-C.); demetrio@colpos.mx (D.S.F.-R.)

[2] Colegio Mexicano de Especialistas en Recursos Naturales AC, De las Flores no. 8 s/n, San Luis Huexotla, Texcoco 56220, State of Mexico, Mexico; arturo.transito@gmail.com

* Correspondence: macedoan@colpos.mx; Tel.: +52-55-2990-6800

Table S1. The sentinel 2 satellite images analyzed in this research work are listed in the table below.

Sentinel 2 satellite images
S2B_MSIL2A_20190303T170209_N0211_R069_T14QMH_20190303T230908
S2A_MSIL2A_20190308T170131_N0211_R069_T14QMH_20190308T225101
S2B_MSIL2A_20190313T170059_N0211_R069_T14QMH_20190313T222930
S2A_MSIL2A_20190318T170021_N0211_R069_T14QMH_20190318T230217
S2B_MSIL2A_20190323T165949_N0211_R069_T14QMH_20190323T224614
S2A_MSIL2A_20190328T170201_N0211_R069_T14QMH_20190328T230437
S2B_MSIL2A_20190402T165849_N0211_R069_T14QMH_20190402T211900
S2A_MSIL2A_20190407T165851_N0211_R069_T14QMH_20190407T230640
S2B_MSIL2A_20190412T165849_N0211_R069_T14QMH_20190412T212318
S2A_MSIL2A_20190417T165901_N0211_R069_T14QMH_20190417T212405
S2B_MSIL2A_20190422T165849_N0211_R069_T14QMH_20190422T224604
S2A_MSIL2A_20190427T165901_N0211_R069_T14QMH_20190427T212400
S2B_MSIL2A_20190502T165859_N0211_R069_T14QMH_20190502T211459
S2A_MSIL2A_20190507T165901_N0212_R069_T14QMH_20190514T162224
S2B_MSIL2A_20190512T165859_N0212_R069_T14QMH_20190512T211647
S2A_MSIL2A_20190517T165901_N0212_R069_T14QMH_20190517T212403
S2B_MSIL2A_20190522T165859_N0212_R069_T14QMH_20190522T205429
S2A_MSIL2A_20190527T165901_N0212_R069_T14QMH_20190527T225955
S2B_MSIL2A_20190601T165859_N0212_R069_T14QMH_20190601T212802
S2A_MSIL2A_20190606T165901_N0212_R069_T14QMH_20190606T230430
S2B_MSIL2A_20190611T165859_N0212_R069_T14QMH_20190611T211441
S2A_MSIL2A_20190616T165901_N0212_R069_T14QMH_20190616T224648
S2B_MSIL2A_20190621T165859_N0212_R069_T14QMH_20190621T211005
S2A_MSIL2A_20190626T165901_N0212_R069_T14QMH_20190626T232154
S2A_MSIL2A_20190706T165901_N0212_R069_T14QMH_20190706T230654
S2B_MSIL2A_20190711T165859_N0213_R069_T14QMH_20190711T211818
S2A_MSIL2A_20190716T165901_N0213_R069_T14QMH_20190716T212559
S2B_MSIL2A_20190721T165859_N0213_R069_T14QMH_20190725T172221
S2B_MSIL2A_20190731T165859_N0213_R069_T14QMH_20190731T224253
S2A_MSIL2A_20190805T165901_N0213_R069_T14QMH_20190805T230255
S2B_MSIL2A_20190810T165849_N0213_R069_T14QMH_20190810T212319
S2A_MSIL2A_20190815T165901_N0213_R069_T14QMH_20190816T003637
S2B_MSIL2A_20190820T165849_N0213_R069_T14QMH_20190820T222322
S2A_MSIL2A_20190825T165851_N0213_R069_T14QMH_20190825T232551
S2B_MSIL2A_20190830T165849_N0213_R069_T14QMH_20190830T211242
S2A_MSIL2A_20190904T165851_N0213_R069_T14QMH_20190904T232805

S2B_MSIL2A_20190919T165949_N0213_R069_T14QMH_20190919T211641
S2A_MSIL2A_20190924T170031_N0213_R069_T14QMH_20190924T211934
S2A_MSIL2A_20191014T170251_N0213_R069_T14QMH_20191014T210736
S2A_MSIL2A_20191024T170401_N0213_R069_T14QMH_20191024T205527
S2A_MSIL2A_20191113T170551_N0213_R069_T14QMH_20191113T212422
